# Arsenic Trioxide Enhances the Efficacy of PD‐1 Inhibitors in Hepatocellular Carcinoma by Inducing Immunogenic Cell Death via the ROS/ERS Pathway

**DOI:** 10.1002/iid3.70214

**Published:** 2025-06-12

**Authors:** Xionghui Wang, Simo Cheng, Yannan Xu, Tianxiao Zheng, Changquan Ling, Juan Du

**Affiliations:** ^1^ School of Traditional Chinese Medicine Naval Medical University Shanghai China; ^2^ Department of Traditional Chinese Medicine First Affiliated Hospital of Naval Medical University Shanghai China; ^3^ Department of Gastroenterology No. 967 Hospital of PLA Joint Logistics Support Force Dalian China; ^4^ No. 920 Hospital of PLA Joint Logistics Support Force Kunming China

**Keywords:** arsenic trioxide, endoplasmic reticulum stress, hepatocellular carcinoma, immunogenic cell death, oxidative stress

## Abstract

**Background:**

Hepatocellular carcinoma (HCC) remains a major global health challenge, with limited efficacy of current immunotherapeutic strategies. Immunogenic cell death (ICD), characterized by the release of damage‐associated molecular patterns (DAMPs), offers a promising approach to enhance antitumor immunity. Arsenic trioxide (ATO), an ICD inducer, may synergize with PD‐1 inhibitors to overcome therapeutic resistance, though the underlying mechanisms remain unclear.

**Methods:**

The cytotoxicity of ATO was evaluated via MTT, clonogenic, and apoptosis assays. ROS levels were quantified using ROS fluorescent probes. ERS activation was confirmed by Western blot detection of Calnexin, PDI, ATF‐4, p‐elF2α, and Caspase‐12. ICD induction was assessed by measuring DAMPs (CRT exposure, HMGB1/ATP/IFN‐β release). The roles of ROS/ERS pathways were dissected using NAC (ROS inhibitor) or 4‐PBA (ERS inhibitor) pre‐treatment. Ex vivo dendritic cell maturation assays analyzed ATO‐treated HCC cells' immunostimulatory capacity, while In Vivo models evaluated immune microenvironment modulation via flow cytometry. Prophylactic/therapeutic tumor vaccine experiments assessed antitumor immunity using ATO‐treated HCC cells as vaccines. Synergy between ATO and PD‐1 blockade was tested in tumor‐bearing mice by combining ATO with anti‐PD‐1 antibodies, monitoring tumor growth kinetics and survival outcomes.

**Results:**

ATO dose‐dependently reduced HCC cell viability while elevating intracellular ROS levels and activating ERS. These processes triggered the release/surface exposure of ICD‐related DAMPs, including CRT, HMGB1, ATP, and IFN‐β, leading to dendritic cells maturation and tumor immune microenvironment remodeling. ATO‐treated HCC cells exhibited enhanced immunogenicity, functioning as prophylactic and therapeutic vaccines to stimulate antitumor immunity. Notably, ATO significantly potentiated the therapeutic efficacy of PD‐1 inhibitors In Vivo.

**Conclusion:**

ATO induces ICD in HCC via a ROS/ERS signaling axis, thereby amplifying antitumor immune responses and synergizing with PD‐1 blockade. These findings support the clinical evaluation of ATO‐PD‐1 inhibitor combinations to improve outcomes in HCC patients.

## 1 Introduction

In 2020, The Lancet reported a staggering 906,000 new cases of hepatocellular carcinoma (HCC) worldwide [1], this highlights the pressing need to improve the diagnosis and treatment of HCC, a significant global health concern. The majority of patients who suffer from HCC are identified when the disease is at an advanced stage [2]. Combined therapy with programmed death 1 (PD‐1) inhibitors and angiogenesis blockers serves as a frontline treatment for advanced HCC [3, 4]. However, many patients exhibit suboptimal responses to immunotherapy [5]. Consequently, it is crucial to explore potential strategies to enhance the efficacy of PD‐1 inhibitors.

Immunogenic cell death (ICD) is a unique mode of cellular death characterized by the ability of ICD to effectively activate the host's immune system, triggering further eradication of tumor cells by the body's immune system [6, 7, 8]. Tumor cells undergoing ICD release or express ICD‐related damage‐associated molecular patterns (DAMPs), such as calreticulin (CRT), high mobility group box‐1 protein (HMGB1), adenosine triphosphate (ATP), and interferon‐β (IFN‐β) [9, 10]. These DAMPs possess potent immunostimulatory functions, inducing the maturation of dendritic cells (DCs) and stimulating the generation of robust antitumor immune responses [11, 12].

Accumulating evidence suggests that resistance to PD‐1 inhibitors is associated with factors such as the immunosuppressive state of the tumor microenvironment [13, 14, 15, 16], inadequate secretion of type I interferons [17, 18], insufficient expression of CRT on tumor cell surfaces [19], and low tumor immunogenicity [10, 20, 21]. Inducing ICD in tumor cells can lead to improvements in the tumor microenvironment [22], increased secretion of type I interferons [23], upregulated CRT expression on tumor cell surfaces [24], enhanced tumor immunogenicity [25] and effectively activate antitumor immunity. Therefore, combining ICD inducers with PD‐1 inhibitors represents a potential approach to improving the efficacy of PD‐1 inhibitors. Multiple studies focusing on cancers such as HCC, prostate cancer, and lung cancer have demonstrated that ICD inducers can effectively enhance the antitumor efficacy of PD‐1 inhibitors by activating antitumor immunity through various pathways [26, 27, 28], validating the ability of ICD inducers to sensitize PD‐1 inhibitors.

**Figure 1:**
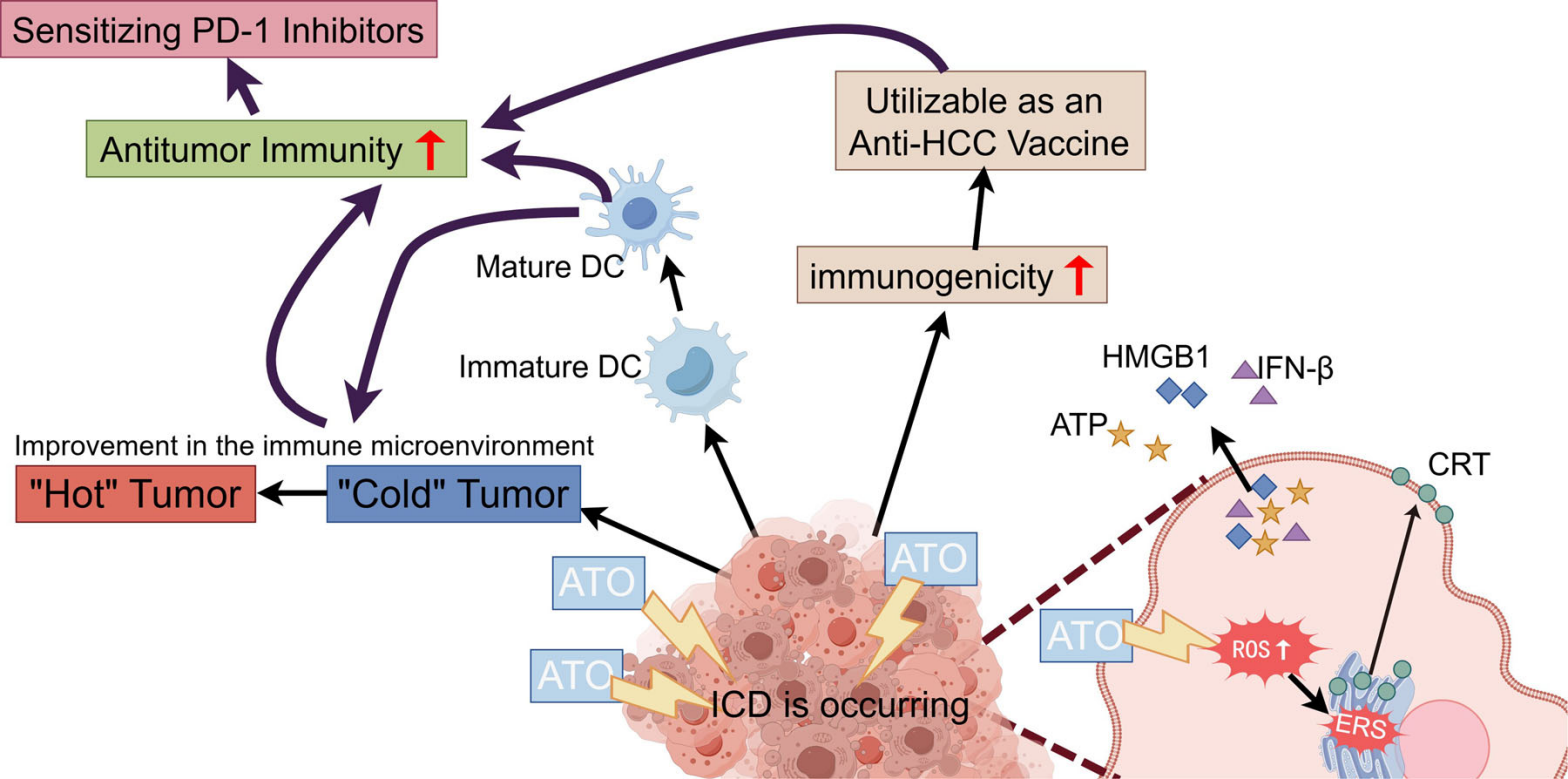
ATO enhanced the efficacy of PD‐1 inhibitors in HCC by inducing ICD via the ROS/ERS pathway.

Arsenic trioxide (ATO), the primary constituent of the traditional Chinese medicine “pishuang” (white arsenic), is recognized as first‐line treatment for advanced HCC by the Chinese Society of Clinical Oncology [3]. The therapeutic potential of ATO in combating HCC has been well‐documented. Studies have shown that ATO effectively prevents the epithelial‐mesenchymal transition (EMT) in HCC cells by upregulating the long noncoding RNA MEG3 through pyruvate kinase M2 (PKM2) [29]. Additionally, ATO triggers autophagy‐accompanied apoptosis, effectively inhibiting tumor growth [30]. Notably, ATO also impedes tumorigenesis by disrupting the paracrine signaling of angiopoietin‐1 (Ang‐1) and angiopoietin‐2 (Ang‐2) [31]. Collectively, these studies provide the evidence for incorporating ATO into the therapeutic arsenal against hepatocellular carcinoma. However, while demonstrating remarkable antitumor efficacy, the clinical application of ATO must consider its dose‐dependent toxicity profile, prolonged exposure to high doses of arsenic compounds is associated with the risk of hepatotoxicity, neurotoxicity, and cardiotoxicity [32, 33]. Research has shown that ATO can trigger ICD in several cancer cells, including HCC [25, 34]. The exact mechanisms are not yet fully understood. Our study demonstrated that ATO induces ICD via the reactive oxygen species (ROS)/endoplasmic reticulum stress (ERS) signaling axis, thereby enhancing antitumor immunity and potentiating the therapeutic efficacy of PD‐1 blockade in HCC (Figure 1).

## 2 Materials and Methods

### 2.1 Cell Cultivation and MTT Assay

The human HCC cell line Huh7 and the mouse HCC cell line Hepa1‐6 were acquired from the Shanghai Cell Bank of the Chinese Academy of Sciences. The cell culture conditions were as previously described [35].

To evaluate cell viability, cells were plated in 96‐well plates at a density of 5 × 10^3^ cells per well and allowed to incubate overnight. Following this, the cells were exposed to various concentrations of ATO (ranging from 0 to 40 μM) for durations of 24, 48, and 72 h. At each designated time point, the MTT assay was performed to evaluate cell viability, following the experimental procedures mentioned earlier [35].

### 2.2 Apoptosis Detection

HCC cells were seeded in a 6‐well plate and cultivated overnight. On the subsequent day, various concentrations of ATO (0, 5, 10, 20 μM) were added to the cultures, which were then incubated for another 24 h. After the incubation period, the measurement of cell apoptosis rate was conducted according to the instructions provided with the Annexin V‐FITC/PI double staining apoptosis detection kit (Jiancheng Bioengineering, Nanjing, China) [36].

### 2.3 Clonal Formation Assay

Each well of a 6‐well plate was seeded with a suspension containing 800 HCC cells in 1 mL. Once the cells adhered to the plate, they were exposed to ATO at concentrations of 0, 5, 10, and 20 μM for 24 h. Subsequently, the ATO‐containing medium was replaced with fresh, complete medium devoid of ATO, and the cells were allowed to grow for an additional 14 days to facilitate colony formation. When visible colonies formed, growth was halted, and the cells were fixed using 4% paraformaldehyde (Beyotime, Shanghai, China) for 10 min. After washing with PBS, the colonies were stained with crystal violet (Beyotime, Shanghai, China) for 15 min. Following PBS rinse, the plates were allowed to air‐dry and were then photographed for documentation. The percentage of colony area was determined using ImageJ analysis software.

### 2.4 Detection of ROS

HCC cells were grown in 6‐well plates overnight and then exposed to various concentrations of ATO (0, 5, 10, and 20 μM) for 24 h. Alternatively, the cells were pretreated with 5mM N‐acetylcysteine (NAC, Beyotime, Shanghai, China) for 2 h before the addition of 20 μM ATO for another 24 h. After the treatment period, the levels of ROS within the cells were then quantified according to the instructions provided with the ROS detection kit (Beyotime, Shanghai, China) [36].

### 2.5 Western Blot Analysis

Protein used for Western blot analysis was extracted from HCC cell lines, and it was extracted by RIPA (Beyotime, Shanghai, China) and protease inhibitor cocktail (Epizyme, Shanghai, China) at the ratio of 100:1. After the extraction of proteins, a Western blot analysis was performed, adhering to the established protocols [35]. The following proteins were analyzed using the Western blot technique: Calnexin (CST, Massachusetts, USA), PDI (CST, Massachusetts, USA), p‐elF2α (CST, Massachusetts, USA), Caspase 12 (Abcam, Cambridge, UK), β‐actin (CST, Massachusetts, USA), ATF‐4 (Santa Cruz Biotechnology, Shanghai, China) and GAPDH (CST, Massachusetts, USA). The dilution ratio of the primary antibodies used in the Western blotting analysis analysis was 1:1000.

### 2.6 Assay for Cell Membrane CRT Protein Expression

4 × 10^5^ HCC cells were seeded into a six‐well plate. Once the cells had adhered to the plate, they were exposed to various concentrations of ATO (0, 10, 15, and 20 μM). Alternatively, the cells were pretreated with 5 mM NAC for 2 h before being subjected to 20 μM ATO for 24 h. In another scenario, the cells were pretreated with 2 mM 4‐phenylbutyric acid (4‐PBA, Selleck, Texas, USA) for 2 h before the addition of 20 μM ATO for 24 h. After the treatment period, the cells were digested, collected, and transferred into a centrifuge tube. They were then centrifuged, and the resulting cell pellet was blocked with 2% bovine serum albumin (Beyotime, Shanghai, China) for 15 min. Following the blocking step, CRT primary antibody (CST, Massachusetts, USA, 1:600) was added, and the cells were incubated at room temperature for 45 min. After incubation, the cells were washed with PBS. Subsequently, a diluted secondary antibody (Fluor488‐conjugated, Affinity Biosciences, Suzhou, China, 1:200) was added, and the cells were incubated at room temperature for another 45 min. Following the second incubation, the cells were washed again and resuspended in cell staining buffer. Finally, the prepared sample was analyzed using a flow cytometer to assess the level of CRT expression on the cell surface.

### 2.7 Detection of Extracellular HMGB1 and IFN‐β

Initially, 5 × 10^4^ Huh7 or Hepa1‐6 cells were seeded into a 12‐well dish and incubated overnight to allow them to adhere. Once attached, the cells were exposed to various concentrations of ATO (0, 10, 15, and 20 μM). Alternatively, the cells were pretreated with 5 mM NAC for 2 h before being subjected to 20 μM ATO for 24 h. In another scenario, the cells were pretreated with 2 mM 4‐PBA for 2 h before the addition of 20 μM ATO for 24 h. After the treatment period, the supernatant was gently aspirated and transferred into 1.5 mL tubes. These tubes were then centrifuged at 4°C and 13,000 rpm for 10 min. Following centrifugation, the levels of extracellular HMGB1 and IFN‐β were measured using the HMGB1 Elisa Kit (Elabscience, Wuhan, China) and the IFN‐β Elisa Kit (Solarbio, Beijing, China), respectively, according to the manufacturers' protocols.

### 2.8 ATP Measurement

Initially, 5 × 10^4^ HCC cells were plated in a 12‐well dish and allowed to attach to the surface. Once adhered, the cells were exposed to various concentrations of ATO (0, 10, 15, 20 μM). Alternatively, the cells were pretreated with 5 mM NAC for 2 h before the addition of 20 μM ATO for 24 h. In another scenario, the cells were pretreated with 2 mM 4‐PBA for 2 h before the addition of 20 μM ATO for 24 h. After the treatment period, the supernatant was collected into 1.5 mL tubes and centrifuged at 13,000 rpm for 10 min. The supernatant, postcentrifugation, was then transferred to fresh tubes and kept on ice as extracellular ATP samples awaiting analysis. Subsequently, 150 μL of lysis buffer was utilized to lyse the cells on ice for 15 min. The lysate was transferred to 1.5 mL tubes and centrifuged again at 13,000 rpm for 10 min. The resulting supernatant, which contained the intracellular ATP, was collected and stored on ice. The ATP content was determined using an ATP detection kit (Beyotime, Shanghai, China) according to the manufacturer's guidelines, with the results measured in relative light units. The protein concentrations in the remaining lysate samples were quantified using a BCA protein assay kit (Thermo Fisher, MA, USA). The ATP concentration was normalized to the protein concentration of each sample, and the final results were reported as nmol/mg protein.

### 2.9 Assay for Dendritic Cell Maturation

Isolation and cultivation of mouse bone Marrow–derived dendritic cells (BMDCs): 4‐week‐old mice were euthanized through cervical dislocation, and their femurs and tibias were collected. These bones were flushed approximately ten times with 1640 medium (Gibco, California, USA) until they turned white. The resultant cell suspension was filtered through a 70 μM cell strainer, centrifuged at 1500 rpm for 6 min, and the supernatant was discarded. To eliminate red blood cells, the cells were treated with a red blood cell lysis buffer (Beyotime, Shanghai, China) and incubated at room temperature for 6 min. After being washed with PBS and centrifuged once again, the cells were cultured in a specialized BMDC medium consisting of 1640 complete medium (Gibco, California, USA) supplemented with recombinant murine granulocyte‐macrophage colony‐stimulating factor (20 ng/mL) (Novoprotein, Suzhou, China) and interleukin‐4 (20 ng/mL) (Novoprotein, Suzhou, China). On Days 4 and 7, half of the culture medium was replaced with fresh BMDC medium. On Day 10, the suspended cells were collected by transferring the medium in the dish to a centrifuge tube, and then the dish was gently rinsed with fresh 1640 medium to harvest the loosely adherent and semi‐adherent cells.

Induction of BMDC Maturation: On Day 9, Hepa1‐6 cells were treated with ATO at various concentrations (0, 10, 15, 20 μM) for 24 h, or they were pretreated with 5 mM NAC for 2 h before being exposed to 20 μM ATO for an additional 24 h. On Day 10, the previously treated Hepa1‐6 cells and the BMDCs collected in the previous step were combined at a 1:1 ratio and cocultured in a six‐well plate for 24 h. Following this coculture, the floating cells were collected, and the plate was gently rinsed to gather the semi‐adherent cells. These cells were centrifuged at 1500 rpm for 6 min. The cells were then incubated with an anti‐mouse CD16/32 antibody (BioLegend, CA, USA, 1:1000) for 20 min to block nonspecific binding. Subsequently, antibodies against CD11c (BioLegend, CA, USA, 1:1000), CD80 (BioLegend, CA, USA, 1:1000), and CD86 (BioLegend, CA, USA, 1:1000) were added. After incubating for 30 min at 4°C, the cells were washed with PBS and analyzed using flow cytometry.

### 2.10 Establishment and Intervention of Mouse Subcutaneous HCC Models

To establish mouse subcutaneous HCC models, Hepa1‐6 cells (100 μL, containing 2 × 10^6^ cells) or H22 cells (100 μL, containing 1 × 10^6^ cells) were injected into the inguinal area. Once the average tumor volume reached 100 mm³, the mice were randomly assigned to various treatment groups. The control group received daily intraperitoneal injections of 100 μL of saline. The ATO group was administered daily intraperitoneal injections of 4 mg/kg of ATO per mouse (in a 100 μL volume). The PD‐1 group received intraperitoneal injections of anti‐mouse PD‐1 (BioXCell, New Hampshire, USA) every 3 days at a dosage of 10 mg/kg per mouse (in a 100 μL volume). The ATO + PD‐1 group received both ATO and anti‐mouse PD‐1 according to the aforementioned doses and schedules. The mice's health status, tumor size, and body weight were monitored throughout the study. Mice with Hepa1‐6 tumors were euthanized between Days 10 and 11 of treatment, while those with H22 tumors were observed until their tumors reached or exceeded 2000 mm³, at which point their survival durations were recorded.

### 2.11 In Vivo Investigation to Evaluate the Prophylactic and Therapeutic Tumor Vaccine

Prophylactic Vaccine Experiment: Hepa1‐6 cells were treated with 20 μM ATO for 24 h. After this period, the supernatant and cells were collected, centrifuged, and then washed with PBS. The cells were counted and adjusted to a concentration of 2 × 10^7^ cells per milliliter to create the HCC prophylactic tumor vaccine. Mice in the ATO group received a 100 μL injection of this vaccine (containing 2 × 10^6^ cells) in the left inguinal area, while the control group received an equivalent volume of PBS. At Days 7 and 40 postvaccination, both groups were “rechallenged” with a 100 μL injection of untreated Hepa1‐6 cell suspension (containing 2 × 10^6^ cells) into the right inguinal area. The mice were continuously monitored for tumor development, weight changes, and any unusual behaviors.

Therapeutic Vaccine Experiment: On Day 0, mice were injected with a 100 μL suspension of untreated Hepa1‐6 cells (containing 2 × 10^6^ cells) in the right inguinal area. Subsequently, Hepa1‐6 cells were treated with 20 μM ATO for 24 h, and the supernatant and cells were harvested, centrifuged, washed with PBS, and counted to prepare the therapeutic tumor vaccine at a concentration of 2 × 10^7^ cells per milliliter. On Day 3, mice in the ATO group received a 100 μL injection of the therapeutic vaccine (containing 2 × 10^6^ cells) in the left inguinal area, while the control group received an equivalent volume of PBS. The mice were monitored for tumor progression in the right inguinal area, fluctuations in body weight, and any abnormal behaviors. Tumor volume was calculated using the formula *V* = 0.5 × *L* × *W* ^ 2, where *V* represents the subcutaneous tumor volume, *L* was the tumor length, and *W* was the tumor width.

### 2.12 Detection of Tumor‐Infiltrating Immune Cells

Eleven days posttreatment with either ATO or PBS, the mice underwent euthanasia via cervical dislocation. Subsequently, the subcutaneous Hepa1‐6 tumor tissues were excised and immersed in a digestion solution composed of collagenase IV (1 mg/mL; Invitrogen, CA, USA) and DNase I (1 μg/mL; Sigma‐Aldrich, MO, USA). The tissues were meticulously minced and incubated in a shaking incubator maintained at 37°C and 80 rpm for 90min, with intermittent inversion of the tube every 10 min to facilitate digestion, ultimately yielding a single‐cell suspension. Postprocessing, the lysate was filtered through a 70 μM mesh, and the filtrate was transferred to a fresh tube. Centrifugation was performed to discard the supernatant, followed by washing the cells with PBS. A red blood cell lysis buffer (Beyotime, Shanghai, China) was introduced and incubated at room temperature for 6 min to eliminate red blood cells. After centrifugation, the supernatant was removed, and the cells were washed again with PBS. The cells were then suspended in PBS and then blocked using an anti‐mouse CD16/32 antibody (BioLegend, CA, USA; 1:1000) and subsequently stained with the Zombie Aqua Fixable Viability Kit (BioLegend, CA, USA) for 20 min to differentiate live and dead cells. Specific antibodies were then added to each tube for staining purposes. The following antibodies were used: CD45 (BioLegend, CA, USA; 1:1000), CD3 (BioLegend, CA, USA; 1:1000), CD8a (BioLegend, CA, USA; 1:1000), CD11b (BioLegend, CA, USA; 1:100), and Ly‐6G/Ly‐6C (Gr‐1; BioLegend, CA, USA; 1:1000).

The cell type analysis was conducted using a CytoFlex flow cytometer (Beckman Coulter, CA, USA), and the data were analyzed with FlowJo software (version 10.8.1; BD Biosciences, New Jersey, USA).

### 2.13 Statistical Analysis

The analysis of data was conducted utilizing SPSS Statistics version 27.0 software, provided by IBM (Armonk, NY, USA). The data presented in this study were presented as mean ± standard deviation (SD). Statistical comparisons to the control group were performed using student's t‐test and the analysis of differences in survival curves employed the log‐rank test method. A *p* value less than 0.05 was deemed indicative of statistical significance.

## 3 Results

### 3.1 ATO Inhibited the Viability of HCC Cells

**Figure 2:**
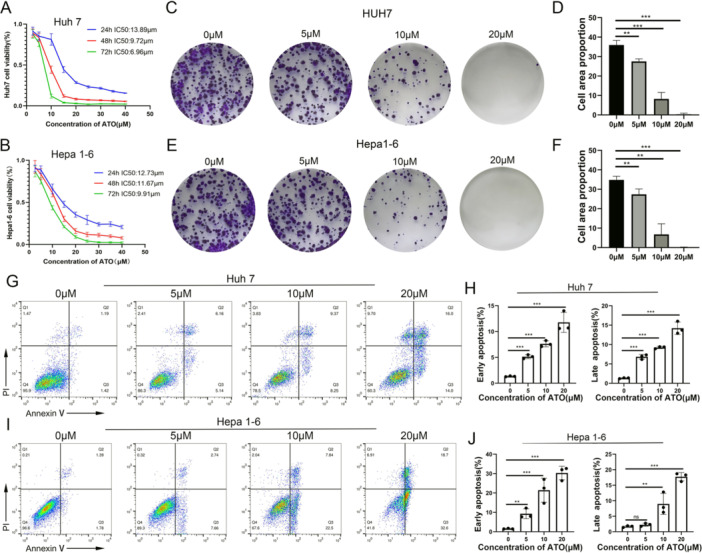
ATO inhibited the viability of HCC cells. (A) Drug dose‐cell viability curves of Huh7 cells treated with ATO for 24, 48, and 72 h. (B) Drug dose‐cell viability curves of Hepa1‐6 cells treated with ATO for 24, 48, and 72 h. (C) Representative clonal images of Huh7 cells at various concentrations of ATO after 24 h treatment. (D) Ratio of Huh7 cells clone area to culture area (one well of a six‐well plate) (*n* = 3). (E) Representative clonal images of Hepa1‐6 cells at various concentrations of ATO after 24 h treatment. (F) Ratio of Hepa1‐6 cells clone area to culture area (one well of a six‐well plate) (*n* = 3). (G) Representative flow cytometry images of Huh7 cells at various concentrations of ATO after 24 h treatment. (H) Proportion of early apoptotic Huh7 cells and late apoptotic Huh7 cells (*n* = 3). (I) Representative flow cytometry images of Hepa1‐6 cells at various concentrations of ATO after 24 h treatment. (J) Proportion of early apoptotic Hepa1‐6 cells and late apoptotic Hepa1‐6 cells (*n* = 3). Data are presented as mean ± standard deviation. ns: no statistical significance; **p < 0.01; ***p < 0.001.

We examined the impact of ATO on HCC cells using three distinct assays: the MTT assay, the clonal formation assay, and the apoptosis assay. Our findings showed that ATO suppressed the growth of HCC cells, with the effect being both concentration‐ and time‐dependent. For Huh7 cells, the half‐maximal inhibitory concentration (IC50) values at 24, 48, and 72 h were 13.98, 9.72, and 6.96 μM, respectively. For Hepa1‐6 cells, the IC50 values were 12.73, 11.67, and 9.91 μM, respectively, as depicted in Figure 2A,B. The clonal formation assay indicated that ATO reduced the ability of both Huh7 and Hepa1‐6 cells to form colonies, with this reduction being proportional to the ATO concentration (Figure 2C–F). Additionally, ATO induced apoptosis in HCC cells in a dose‐dependent fashion. Both the early (Annexin V^+^/PI^−^) and late (Annexin V^+^/PI^+^) stages of apoptosis were observed to increase with rising concentrations of ATO (Figure 2G–J).

### 3.2 ATO Triggers ROS Production and ERS in HCC Cells

**Figure 3:**
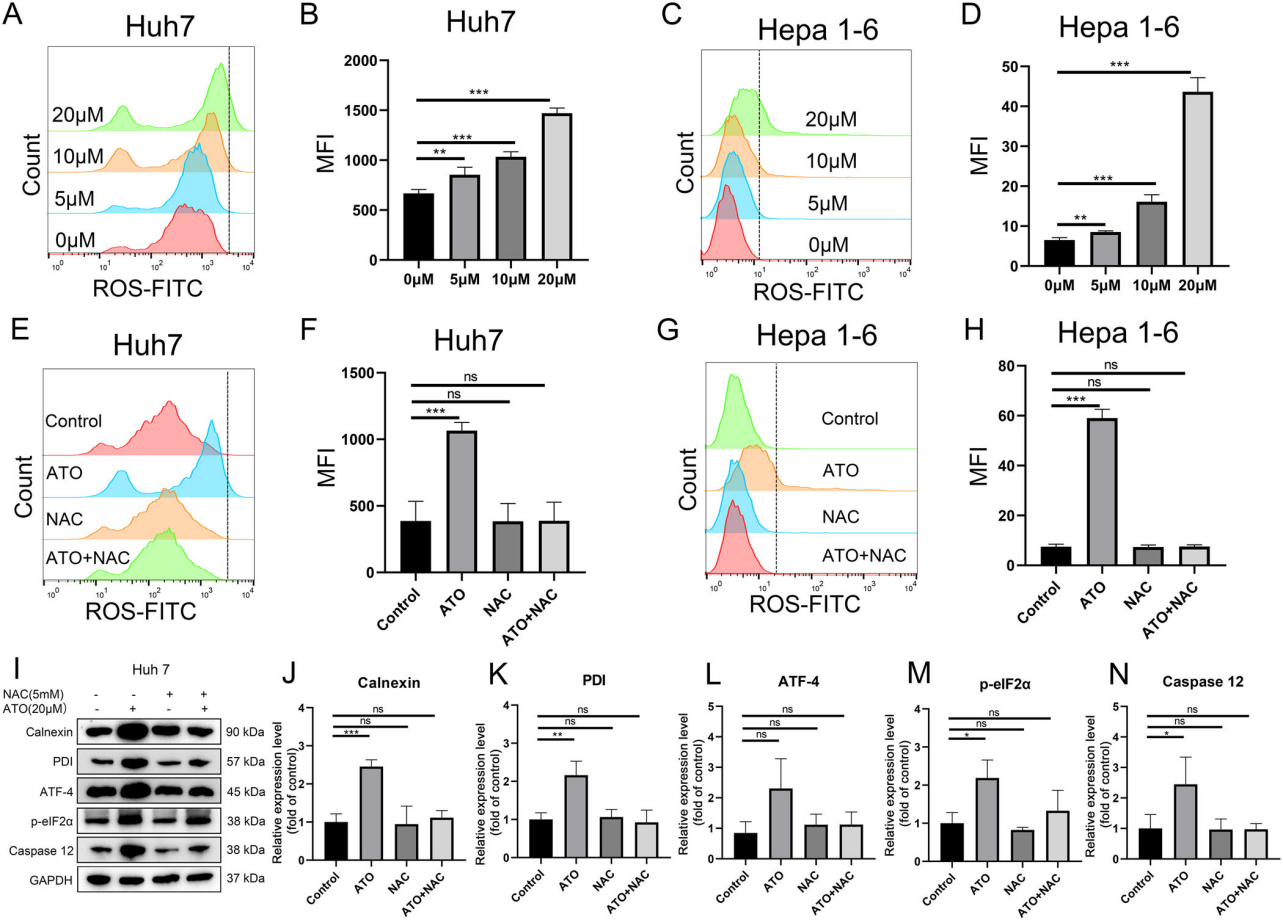
ATO triggers ROS production and ERS in HCC cells. Representative ROS‐FITC fluorescence intensity histograms (A) and bar graphs of mean fluorescence intensity (MFI) (B) of Huh7 cells treated with different concentrations of ATO for 24 h. Representative ROS‐FITC fluorescence intensity histograms (C) and bar graphs of MFI (D) of Hepa1‐6 cells treated with different concentrations of ATO for 24 h. Representative ROS‐FITC fluorescence intensity histograms (E) and bar graphs of MFI (F) of Huh7 cells treated with 20 μM ATO and/or NAC for 24 h. Representative ROS‐FITC fluorescence intensity histograms (G) and bar graphs of MFI (H) of Hepa1‐6 cells treated with 20 μM ATO and/or NAC for 24 h. Representative Western blot images of ERS‐related proteins in Huh7 cells (I). ImageJ software was used to analyze the grayscale values of Calnexin (J), PDI (K), ATF‐4 (L), p‐eIF2α (M), and Caspase 12 (N) proteins (*n* = 3). *p < 0.05; **p < 0.01; ***p < 0.001.

Additionally, our findings revealed that ATO triggered a concentration‐dependent increase in ROS within Huh7 and Hepa1‐6 cells (Figure 3A–D). This increase in ROS levels was mitigated by the addition of NAC (a ROS inhibitor) (Figure 3E–H). Previous studies have suggested a potential correlation between intracellular ROS accumulation and ERS [36]. In this study we utilized the western blot to investigate the expression of ERS‐associated proteins, including Calnexin, PDI, ATF‐4, phosphorylated eIF2α, and Caspase 12, in ATO‐treated HCC cells. The data showed that ATO significantly enhanced the expression of these ERS‐associated proteins, an effect that was markedly attenuated by inhibiting ROS production (Figure 3I–N). This suggests that ATO induces ERS in HCC cells through a ROS‐dependent pathway.

### 3.3 ATO Induced ROS/ERS‐Dependent Damps

**Figure 4:**
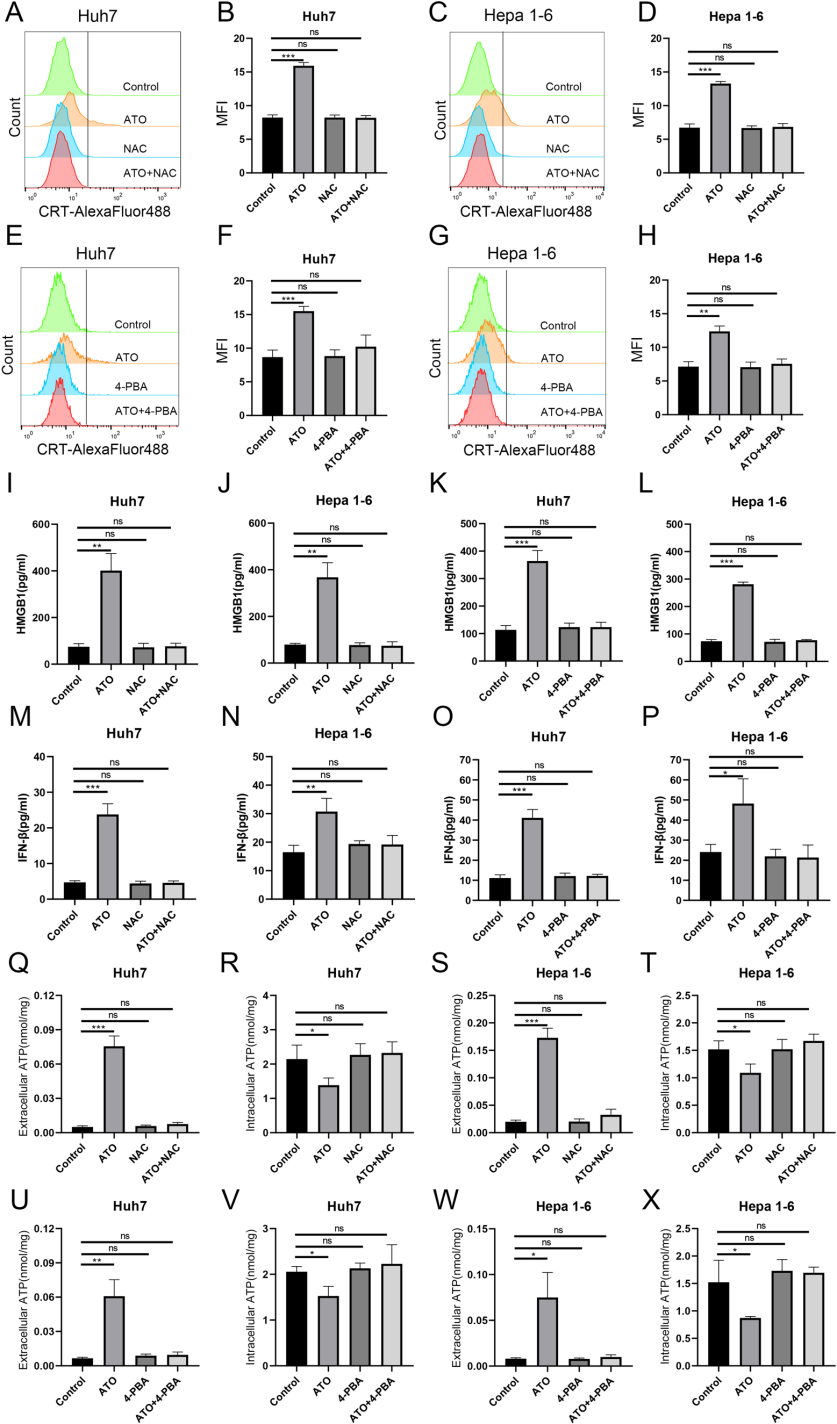
ATO induced ICD‐related DAMPs through ROS/ERS pathway. Representative CRT‐AlexaFluor488 fluorescence intensity histograms (A) and bar graphs of MFI (B) of Huh7 cells treated with ATO and/or NAC for 24 h. Representative CRT‐AlexaFluor488 fluorescence intensity histograms (C) and bar graphs of MFI (D) of Hepa1‐6 cells treated with ATO and/or NAC for 24 h. Representative CRT‐AlexaFluor488 fluorescence intensity histograms (E) and bar graphs of MFI (F) of Huh7 cells treated with ATO and/or 4‐PBA for 24 h. Representative CRT‐AlexaFluor488 fluorescence intensity histograms (G) and bar graphs of MFI (H) of Hepa1‐6 cells treated with ATO and/or 4‐PBA for 24 h. Extracellular HMGB1 levels in Huh7 cells (I) or Hepa1‐6 cells (J) after treatment with ATO and/or NAC. Extracellular HMGB1 levels in Huh7 cells (K) or Hepa1‐6 cells (L) after treatment with ATO and/or 4‐PBA. Extracellular IFN‐β levels in Huh7 cells (M) or Hepa1‐6 cells (N) after treatment with ATO and/or NAC. Extracellular IFN‐β levels in Huh7 cells (O) or Hepa1‐6 cells (P) after treatment with ATO and/or 4‐PBA. Extracellular ATP content (Q) and intracellular ATP content (R) of Huh7 cells after ATO and/or NAC treatment. Extracellular ATP content (S) and intracellular ATP content (T) of Hepa1‐6 cells after ATO and/or NAC treatment. Extracellular ATP content (U) and intracellular ATP content (V) of Huh7 cells after ATO and/or 4‐PBA treatment. Extracellular ATP content (W) and intracellular ATP content (X) of Hepa1‐6 cells after ATO and/or 4‐PBA treatment. *p < 0.05; **p < 0.01; ***p < 0.001.

Our study showed that ATO induced ICD‐related DAMPs in a dose‐dependent fashion in both Huh7 and Hepa1‐6 cells. This included the exposure of CRT protein on the cell surface (Supporting Information S1: Figure S1A–D), the secretion of HMGB1 (Supporting Information S1: Figure S1E,F), the release of interferon‐β (IFN‐β) (Figure S1G,H), and alterations in ATP secretion and intracellular ATP levels (Supporting Information S1: Figure S1I–L). Pretreatment with NAC (a ROS inhibitor) or 4‐PBA (an ERS inhibitor) to ATO administration abrogated the induction of ICD‐related DAMPs (Figure 4A–X). The levels of CRT (Figure 4A–H), HMGB1 (Figure 4I–L), IFN‐β (Figure 4M–P), and ATP (Figure 4Q–X) in the ATO + NAC group and the ATO + 4‐PBA group were comparable to those in the control group, and these differences were not statistically significant, suggesting that ATO‐induced ICD is dependent on the ROS/ERS pathway.

### 3.4 The Ability of HCC Cells to Induce Dentritic Cells Maturation was Enhanced After ATO Treatment

**Figure 5:**
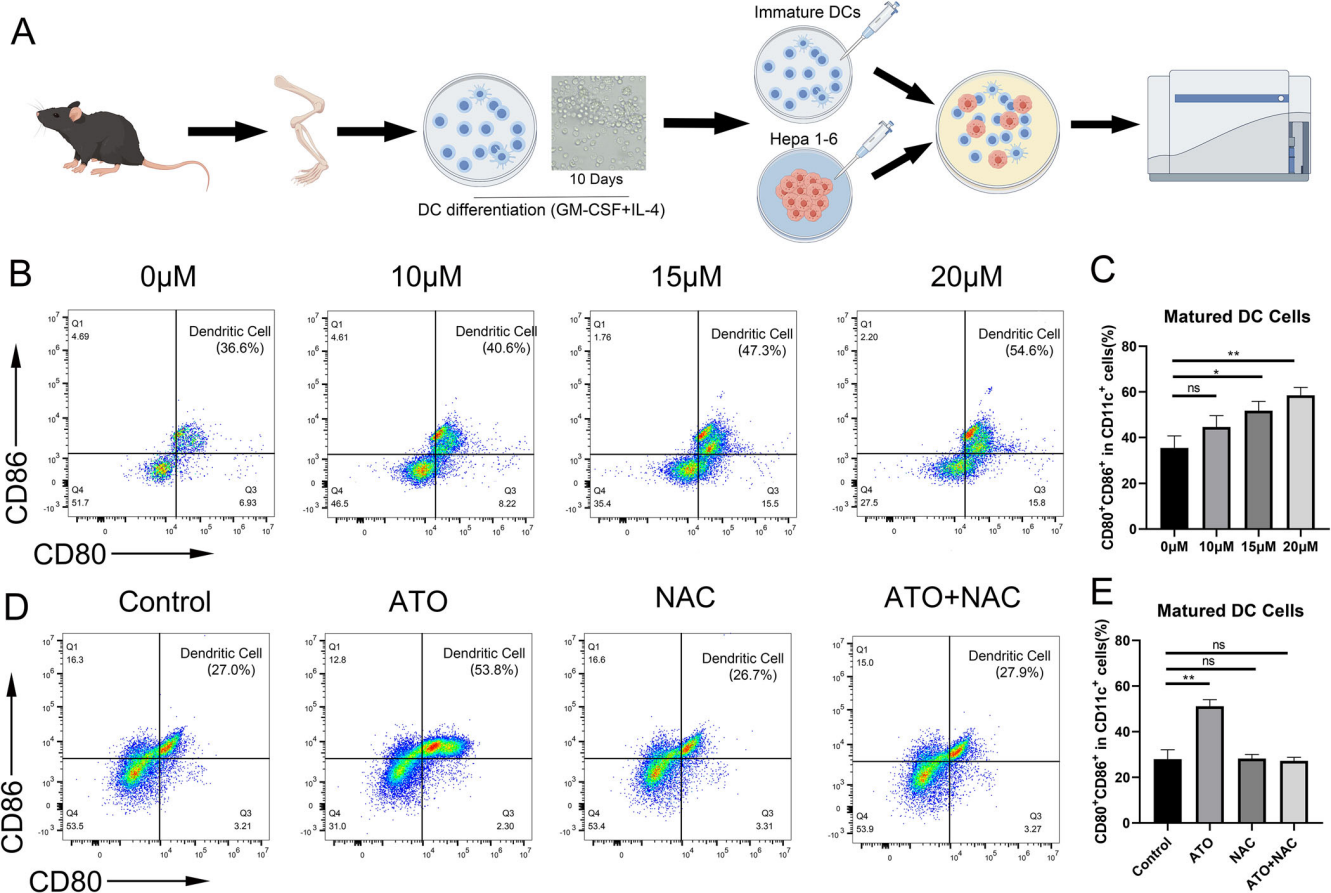
The ability of HCC cells to induce DC maturation was enhanced after ATO treatment. (A) Flowchart of the experimental procedure for DC maturation assays. (B) Representative flow cytometry detection results for the concentration groups of 0, 5, 10, and 20 μM. (C) DC maturation rate (the percentage of CD80^+^CD86^+^ cells among CD11c^+^ cells) (*n* = 3). (D) Representative flow cytometry detection results for the control group, ATO group, NAC group, and ATO + NAC group. (E) DC maturation rate (the percentage of CD80^+^CD86^+^ cells among CD11c^+^ cells) (*n* = 3). Each bar represents the mean of three replicates. ns: not statistically significant, *p < 0.05, **p < 0.01.

ICD‐related DAMPs can serve as an “adjuvant” to enhance immune responses by potently stimulating dendritic cell maturation [10]. Hepa1‐6 cells were exposed to ATO and cocultured with bone marrow–derived immature DCs (Figure 5A). The level of ICD in Hepa1‐6 cells was gauged by determining the percentage of mature DCs. The findings revealed that the percentage of mature DCs rose in tandem with increasing ATO concentrations (Figure 5B,C). Moreover, the application of NAC reversed the capacity of ATO‐treated Hepa1‐6 cells to promote DC maturation (Figure 5D–F).

### 3.5 ATO Displays In Vivo Vaccine‐Like Antitumor Activity Against HCC

**Figure 6:**
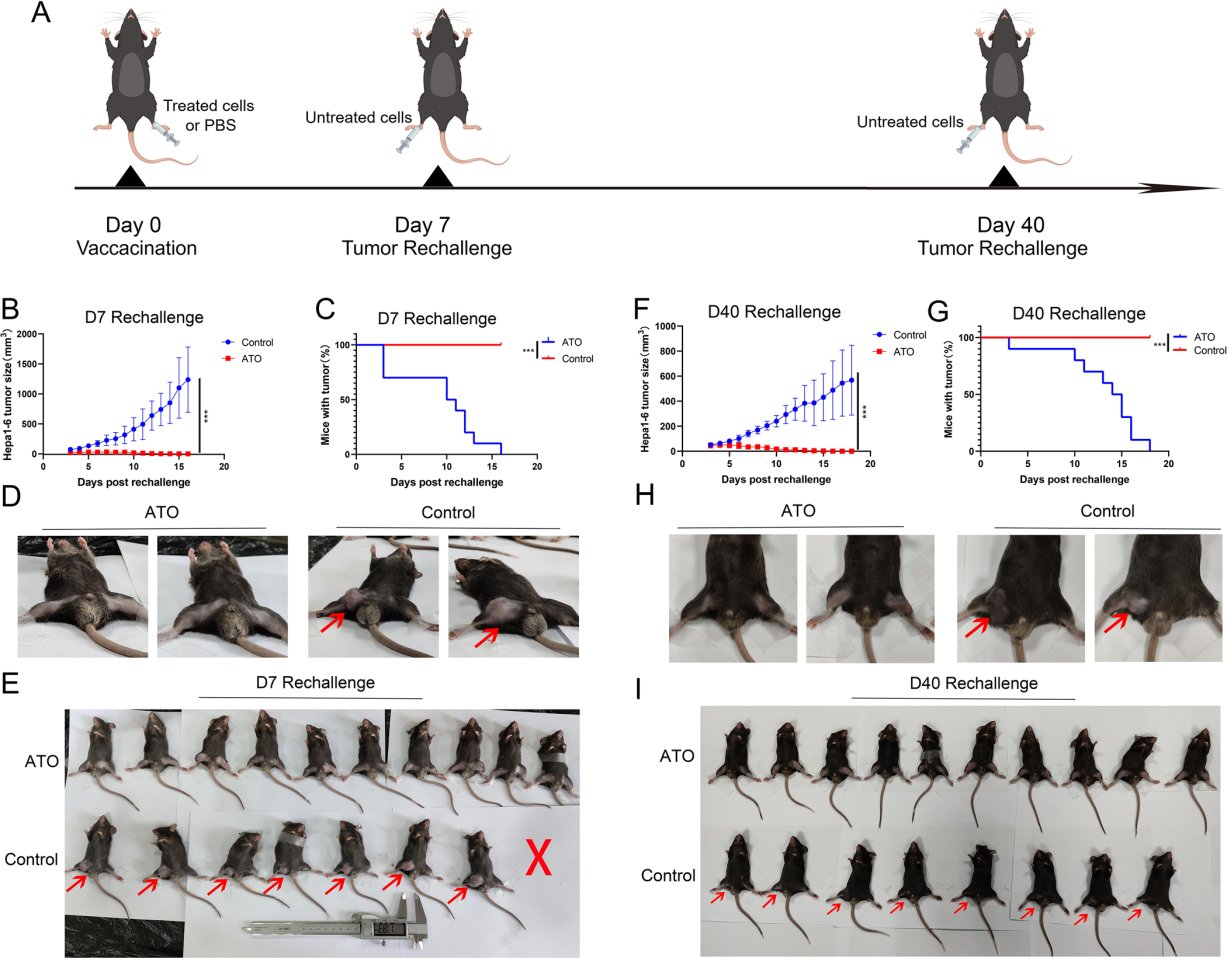
ATO‐treated HCC cells exhibited both short‐term and long‐term anti‐HCC vaccine‐like efficacy In Vivo. (A) Flowchart of the experimental procedure for the prophylactic tumor vaccine. (B) Growth curve of tumor volumes in short‐term prophylactic tumor vaccines experiment. (C) Observation days post “D7 Rechallenge”‐ Plot of the proportion of tumor‐bearing mice. (D) Representative images captured from the inguinal regions of mice in the ATO and control groups on day 16 post “D7 Rechallenge.” (E) Images taken of all mice on Day 16 post “D7 Rechallenge,” where red arrows indicate the presence of Hepa1‐6 tumor tissue, and red “x” mark the mice that were euthanized by cervical dislocation beforehand due to tumor volumes exceeding 2000 mm³. (F) Growth curve of tumor volumes in long‐term prophylactic tumor vaccines experiment. (G) Observation days post “D40 Rechallenge”‐Plot of the proportion of tumor‐bearing mice. (H) Representative images of the inguinal region of mice from the ATO and control groups on Day 17 post “D40 Rechallenge”. (I) Images of all mice on Day 17 post “D40 Rechallenge,” where red arrows indicate the presence of Hepa1‐6 tumor tissue. ***p < 0.001.

The prophylactic tumor vaccines experiment serves as the gold standard for assessing whether tumor cells are subject to ICD. Figure 6A depicted the experimental workflow, where ATO‐treated Hepa1‐6 cells were utilized as a tumor vaccine and injected in the left inguinal area of C57BL/6 mice. The results revealed that tumors in the PBS group continued to grow, whereas the ATO group exhibited a restrained increase in tumor size (Figure 6B). In addition, by day 16 post the “D7 rechallenge,” the PBS group had a 100% tumor occurrence, whereas the tumors in the ATO group had completely resolved. (Figure 6C–E). Similar results were also found in the long‐term prophylactic tumor vaccine experiment. The ATO group exhibited a significant reduction in tumor growth compared to the control group. (Figure 6F). Furthermore, by Day 18 post the “D40 rechallenge,” a 100% tumor occurrence was observed in the PBS group, while the tumors in the ATO group had completely resolved. (Figure 6G–I). The data indicate that ATO has the capacity to activate immune effector cells and initiate an immune memory response, facilitated by the release of DAMPs related to ICD.

**Figure 7:**
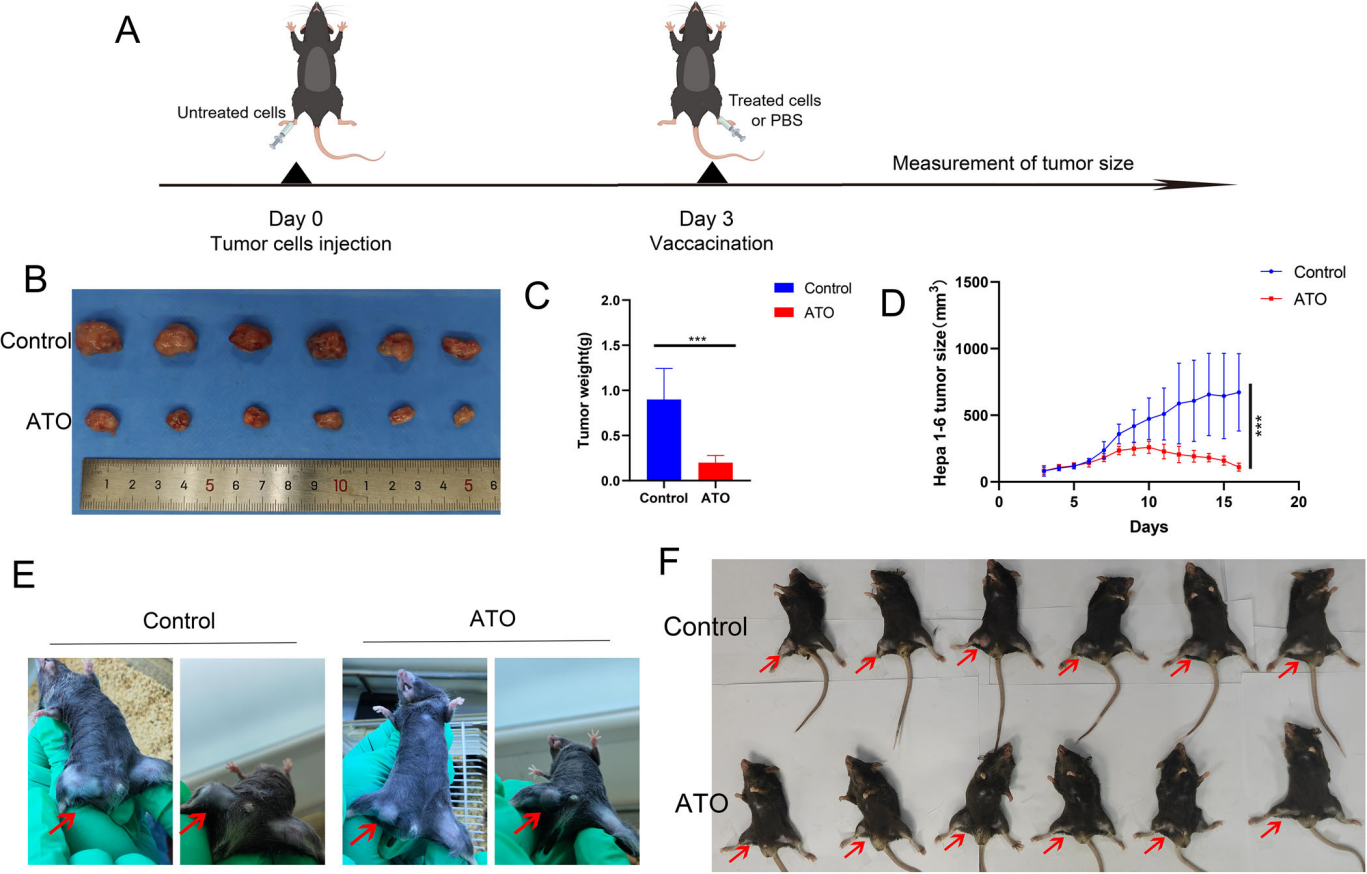
HCC cells treated with ATO can function as a therapeutic tumor vaccine to elicit antitumor immunity. (A) Flowchart of the experimental procedure for the therapeutic tumor vaccine. (B) Growth curve of tumor volumes in the therapeutic tumor vaccine experiment. (C) Images of subcutaneous tumors from various groups. (D) Tumor weights of subcutaneous tumors from various groups. (E) Representative images of the inguinal region of mice from the ATO and control groups on Day 16 of the therapeutic tumor vaccine experiment. (F) Images of all mice on Day 16 of the therapeutic tumor vaccine experiment, where red arrows indicate the presence of Hepa1‐6 tumor tissue. ***p < 0.001.

The therapeutic tumor vaccine experiment is also one of the methods to verify whether tumor cells undergo ICD [10, 25]. The experimental procedure was illustrated in Figure 7A. The results indicated that ATO‐treated Hepa1‐6 cells can serve as effective “therapeutic vaccines” against HCC to elicit antitumor immunity, as evidenced by the decrease in tumor volume and size compared to control (Figure 7B–F).

### 3.6 ATO Improved the Immune Microenvironment In Vivo

**Figure 8:**
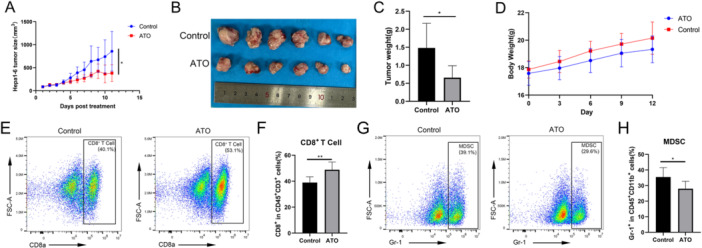
ATO can improve the immune microenvironment of Hepa1‐6 subcutaneous tumors. (A) Average tumor volume change curves during the experiment in control and ATO groups (*n* = 6). (B) Images of Hepa1‐6 subcutaneous tumors. (C) Tumor weights of Hepa1‐6 subcutaneous tumors. (D) Average body weight changes in mice from both groups during the experiment. (E) Representative flow cytometry plots of CD8^+^ T cells in control and ATO groups. (F) Average percentage of CD8^+^ T cells (*n* = 6). (G) Representative flow cytometry plots of myeloid‐derived suppressor cells in control and ATO groups. (H) Average percentage of myeloid‐derived suppressor cells (*n* = 6). ns: no significant difference, *p < 0.05, **p < 0.01.

ATO slowed the growth of subcutaneous Hepa1‐6 tumors in mice, in contrast to the control group treated with PBS (Figure 8A–C). Mouse body weight increased progressively over the course of the experiment (Figure 8D). Flow cytometry revealed that ATO treatment augmented the presence of CD8^+^ T cells (Figure 8E,F) within the tumor microenvironment. Simultaneously, ATO treatment reduced the infiltration of myeloid‐derived suppressor cells (MDSC, Figure 8G,H) within the tumor microenvironment. These changes transformed the “cold” tumor microenvironment into a “hot” one and significantly improved the immune context of HCC.

### 3.7 ATO Enhanced the Anti‐HCC Efficacy of PD‐1 Antibodies In Vivo

**Figure 9:**
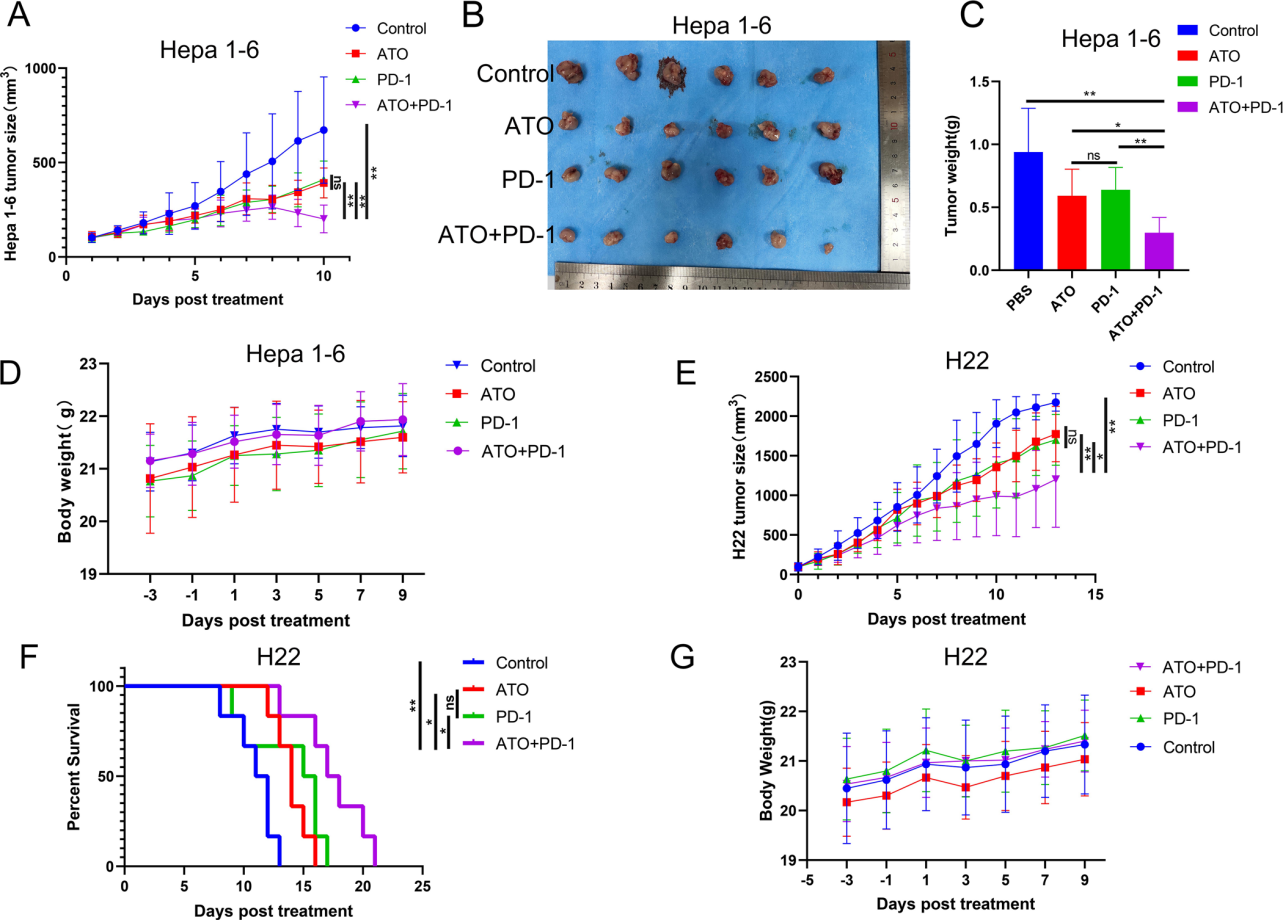
ATO enhances the anti‐HCC efficacy of PD‐1 antibodies In Vivo. (A) Average volume change curves of Hepa1‐6 subcutaneous tumors in different groups of mice during drug administration (*n* = 6). (B) Images of Hepa1‐6 subcutaneous tumors. (C) Weights of Hepa1‐6 subcutaneous tumors. (D) Body weight changes of mice in different groups during drug administration. (E) Volume change curves of H22 subcutaneous tumors in different groups of mice during drug administration (*n* = 6). (F) Survival curves of H22 subcutaneous tumor mouse models in different groups (*n* = 6). (G) Body weight changes of mice in different groups during drug administration. *p < 0.05, **p < 0.01.

The findings revealed that the combined treatment of ATO and PD‐1 inhibitors (ATO + PD‐1 group) was more effective in reducing tumor size and weight in the Hepa1‐6 models than either treatment alone (Figure 9A–C). Throughout the treatment, the mice experienced a steady increase in body weight (Figure 9D). Comparable outcomes were observed in the H22 tumor models, with the ATO + PD‐1 group showing smaller tumor sizes compared to the other groups (Figure 9E). Furthermore, the combined therapy with ATO and PD‐1 inhibitors (ATO + PD‐1 group) significantly prolonged the survival time of the H22 subcutaneous tumor models (Figure 9F). The H22 tumor model mice across all four treatment groups showed a progressive increase in body weight throughout the treatment period (Figure 9G). The data indicates that ATO can significantly improve the therapeutic effectiveness of PD‐1 inhibitors In Vivo.

## 4 Discussion

Given the frequent occurrence of PD‐1 inhibitor resistance in patients with advanced HCC, the medical community has been actively seeking effective solutions. As a kind of ICD inducer, ATO might have the potential to enhance the therapeutic effects of PD‐1 inhibitors in HCC. In this study, we discovered that ATO induces ICD through the ROS/ERS pathway. This process not only directly inhibits tumor cell growth but more importantly activates the antitumor immune response of the body, thereby significantly improving the responsiveness of HCC patients to PD‐1 inhibitors. This discovery explores a new strategy for immunotherapy in HCC and is expected to bring more effective treatment options for patients with advanced HCC.

We firstly observed that ATO not only initiated apoptosis but also stimulated the generation of intracellular ROS. The role of oxidative stress and its implications in tumor immunotherapy are subjects of intense ongoing research [37, 38]. Oxidative stress can activate ERS through various mechanisms [39]. Persistent ERS responses can, in turn, intensify oxidative stress, for instance, by inhibiting glutamine metabolism, decreasing glutathione production, and thus raising intracellular ROS levels; ERS‐induced mitochondrial dysfunction can also produce substantial quantities of ROS [40]. Our findings, which highlight the cytotoxic and immunomodulatory properties of ATO, were consistent with contemporary research on the regulatory functions of ROS and ERS in facilitating cancer cell death and modulating immune reactions [12, 36]. Additionally, we found that ERS induced by ATO in HCC is reliant on ROS.

The activation of ERS is increasingly recognized for its role in provoking antitumor immunity [41, 42]. When ICD inducers trigger ERS‐mediated CRT translocation, the tumor immune microenvironment is often correspondingly improved [42, 43]. This improvement may be one of the mechanisms by which targeting ICD can augment the effectiveness of PD‐1 inhibitors. Our data revealed that ATO can expose CRT on the surface of HCC cells in a ROS/ERS‐dependent fashion, as well as elicit the secretion of HMGB1, ATP, and IFN‐β. Furthermore, ATO‐treated Hepa1‐6 cells effectively induced the maturation of DC, indicating the potential of ATO in bolstering antitumor immunity. Moreover, ATO treatment improved the tumor microenvironment by increasing the infiltration of CD8^+^ T cells and decreasing the infiltration of myeloid‐derived suppressor cells. This transformation effectively converted the “cold” tumor microenvironment into a “hot” one, substantially improving the immune landscape of HCC. Notably, ICD inducers are potent agents capable of significantly increasing the immunogenicity of tumor cells, thereby amplifying the therapeutic benefits of PD‐1 inhibitors [26, 27, 28]. When Hepa1‐6 cells were exposed to ATO, they can function as effective tumor “vaccines,” which is pivotal for stimulating the immune system against HCC. These observations confirmed that ATO can effectively enhance the immunogenicity of tumor cells and activate the body's antitumor immune response, representing a powerful strategy to boost the efficacy of PD‐1 inhibitors.

Future research should aim to delineate the long‐term outcomes of this combined therapy, including the investigation of potential resistance mechanisms and the impact on the immune system. Moreover, exploring the combination of ATO with other immunotherapeutic agents, such as CTLA‐4 inhibitors or T‐cell engaging therapies, may reveal additional synergistic effects.

Two key limitations should be acknowledged in our study. First, while Gr‐1^+^CD11b^+^ cells are commonly used as markers for MDSCs in murine models, it should be explicitly stated that this study did not directly assess the immunosuppressive functionality of these cells through functional assays such as T‐cell proliferation suppression tests. Future studies incorporating functional validation would strengthen the definitive identification of MDSC populations. Second, and equally importantly, our study insufficiently addressed the toxic effects of arsenic trioxide. Given the high toxicity of ATO, it is crucial to ensure its safety while fully leveraging its anticancer activity, balancing its toxicity and efficacy [44]. Existing studies have shown that high doses of arsenic compounds can lead to hepatotoxicity, neurotoxicity, and cardiotoxicity [32, 33]. To address the high toxicity of ATO, some scholars believe that controlling its dosage and employing antidotes can mitigate its toxicity and reduce the side effects associated with ATO. For instance, studies in leukemia patients with a high body weight have indicated that controlling the upper dose limit of ATO at ≤ 10 mg/dose can enhance treatment safety without compromising clinical efficacy. Furthermore, studies have also found that substances such as biochanin A, phloretin, coenzyme Q10, and epigallocatechin‐3‐gallate can significantly reduce the toxic effects of arsenic by alleviating arsenic‐induced oxidative stress and apoptosis [45, 46, 47]. Additionally, targeting the Nrf2‐Keap1 signaling pathway also aids in mitigating the oxidative stress induced by ATO [48]. In light of this, we plan to further conduct research to ascertain the safe dosage of ATO in HCC patients and actively explore more effective methods to reduce its toxicity.

In summary, our study demonstrated that ATO can significantly enhance the therapeutic impact of PD‐1 inhibitors in HCC by inducing ICD. Mechanistically, ATO triggered ICD in a ROS/ERS‐dependent manner. These findings supported the burgeoning evidence advocating for the combination of conventional chemotherapy agents with modern immunotherapies. The potential of ATO as an ICD inducer, along with its synergistic effects when combined with PD‐1 inhibitors, offers a promising approach for improving treatment outcomes in HCC patients.

## 5 Conclusion

In summary, our research has revealed that ATO can induce ICD and improve the tumor immune suppressive microenvironment in HCC, thereby augmenting the efficacy of PD‐1 inhibitors. Importantly, ATO exhibits its potency to induce ICD via a ROS/ERS signaling pathway. Our findings underscore the potential value of further clinical trials examining the synergy between ATO and immune checkpoint inhibitors to enhance therapeutic outcomes in HCC patients.

## Author Contributions

**Xionghui Wang:** validation, formal analysis, investigation, data curation, writing – original draft. **Simo Cheng:** validation, formal analysis, investigation, funding acquisition. **Yannan Xu:** validation, formal analysis, investigation, data curation. **Tianxiao Zheng:** validation, investigation. **Changquan Ling:** conceptualization, resources, writing – review and editing, supervision. **Juan Du:** conceptualization, writing – review and editing, supervision, project administration, funding acquisition.

## Ethics Statement

All experiments related to animal handling were conducted with Shanghai Changhai Hospital Ethics Committee (approval CHEC(A.E)2022‐030).

## Conflicts of Interest

The authors declare no conflicts of interest.

## Supporting information

SuppMat.docx.

## Data Availability

All raw data and code are available upon request.
