# Supplementary material for: Arsenic Trioxide Enhances the Efficacy of PD‐1 Inhibitors in Hepatocellular Carcinoma by Inducing Immunogenic Cell Death via the ROS/ERS Pathway
Source: Immun Inflamm Dis. 2025 Jun 12;13(6):e70214. doi: 10.1002/iid3.70214 (PMC12160338; doi:10.1002/iid3.70214)
Supplement: Supplementary file 1 — SuppMat.docx. [file IID3-13-e70214-s001.docx]

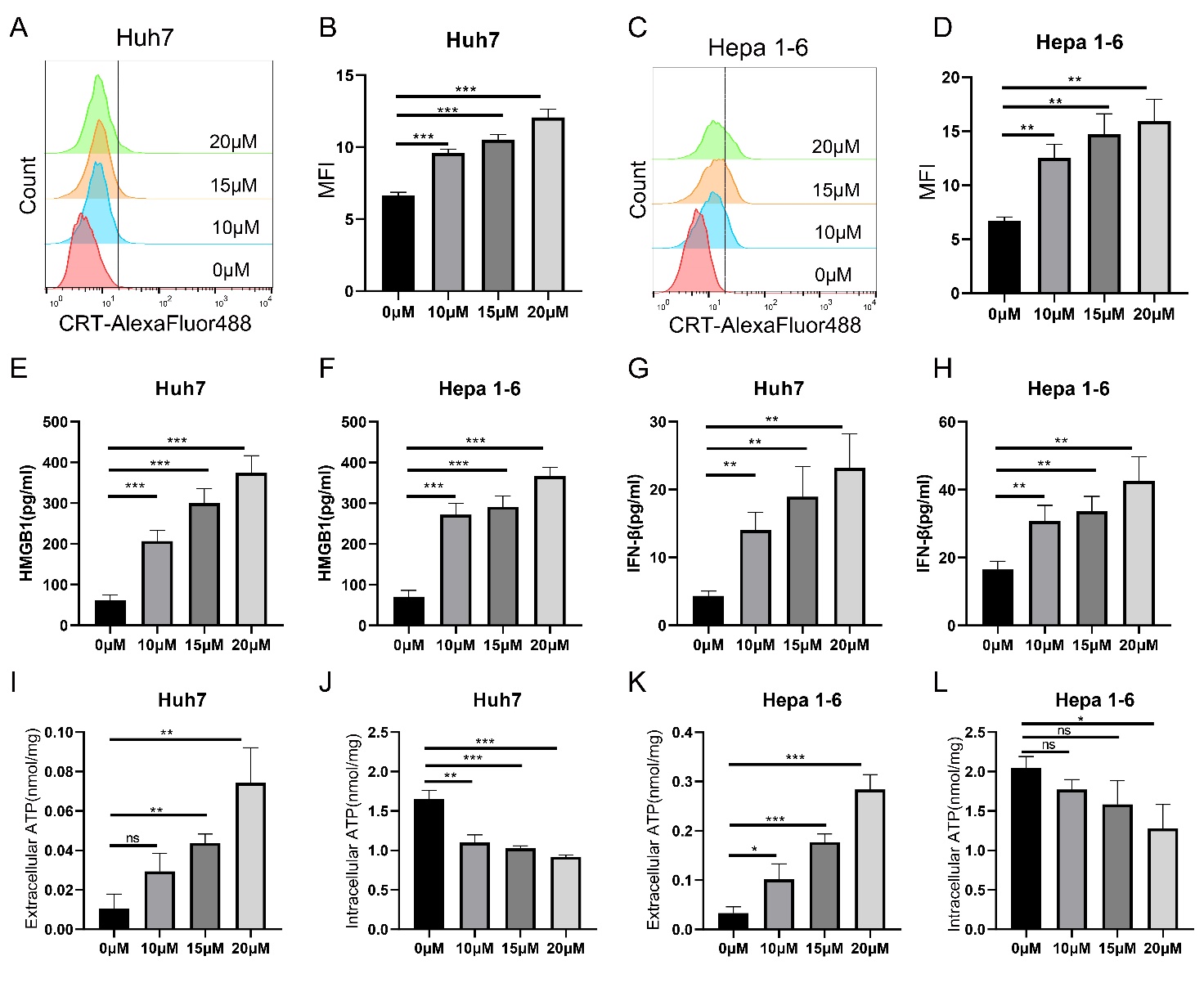


**Fig. S1.** ATO Induced ICD-Related DAMPs in a Concentration-Dependent Manner. Representative CRT-AlexaFluor488 fluorescence intensity histograms (A) and bar graphs of MFI (B) of Huh7 cells treated with different concentrations of ATO for 24 h. Representative CRT-AlexaFluor488 fluorescence intensity histograms (C) and bar graphs of MFI (D) of Hepa1-6 cells treated with different concentrations of ATO for 24 h. Extracellular HMGB1 levels in Huh7 (E) and Hepa1-6 (F) cells after treatment with different concentrations of ATO for 24 h. Extracellular IFN-β levels in Huh7 (G) and Hepa1-6 (H) cells after treatment with different concentrations of ATO for 24 h. Extracellular ATP levels (I) and intracellular ATP levels (J) in Huh7 cells after treatment with various concentrations of ATO for 24 h; extracellular ATP levels (K) and intracellular ATP levels (L) in Hepa1-6 cells after treatment with various concentrations of ATO for 24 h. Each bar represents the mean of three replicates. Data are presented as mean ± standard deviation. ns: not statistically significant; *P < 0.05; **P < 0.01; ***P < 0.001.


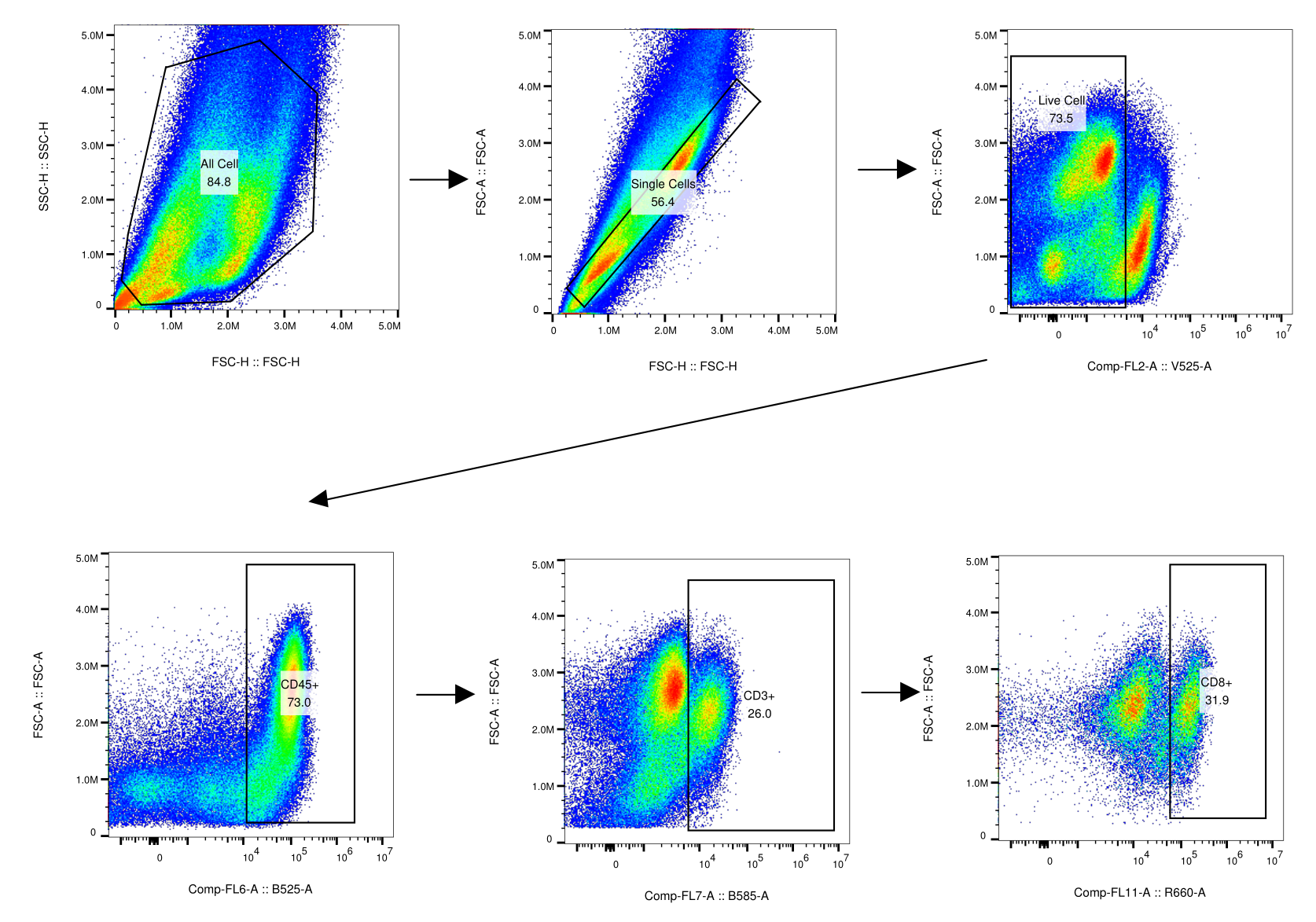


Gating strategy of CD8^+^ T cells


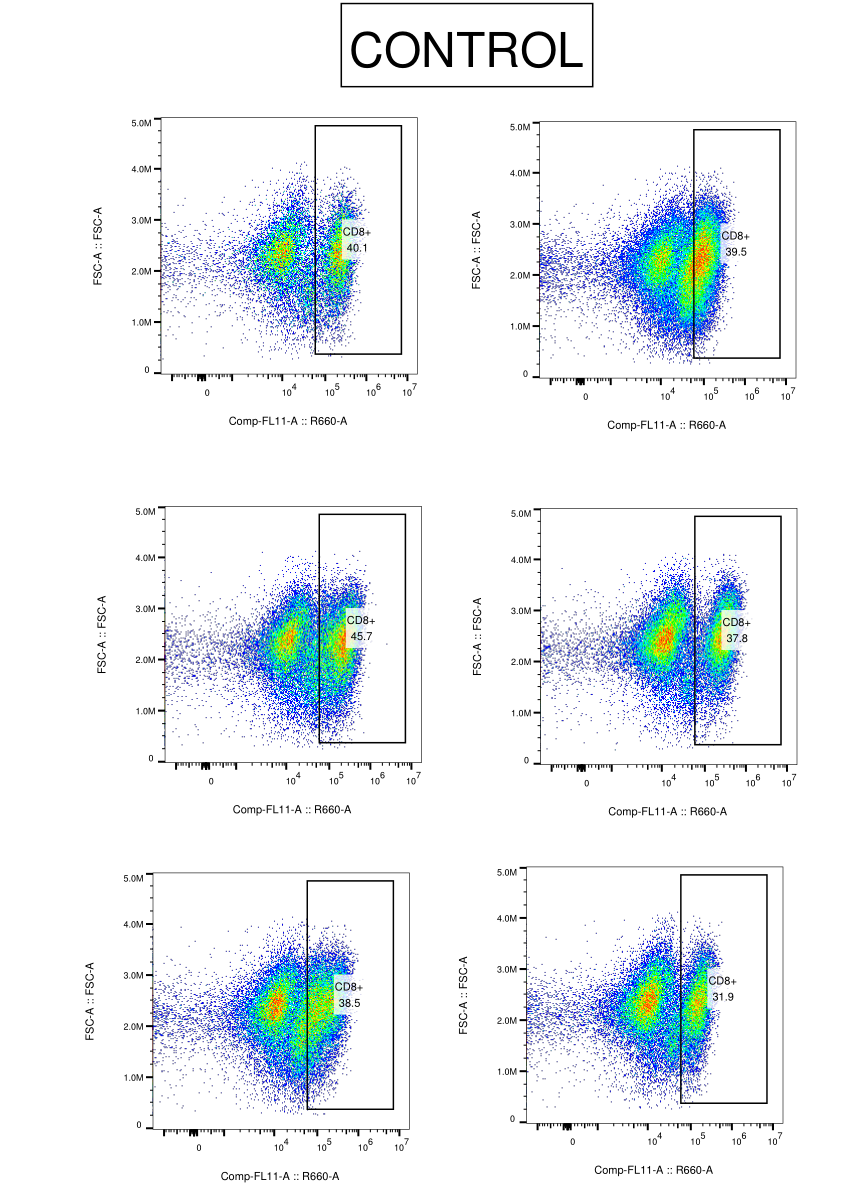


Experimental data of CD8^+^ T cells-CONTROL group


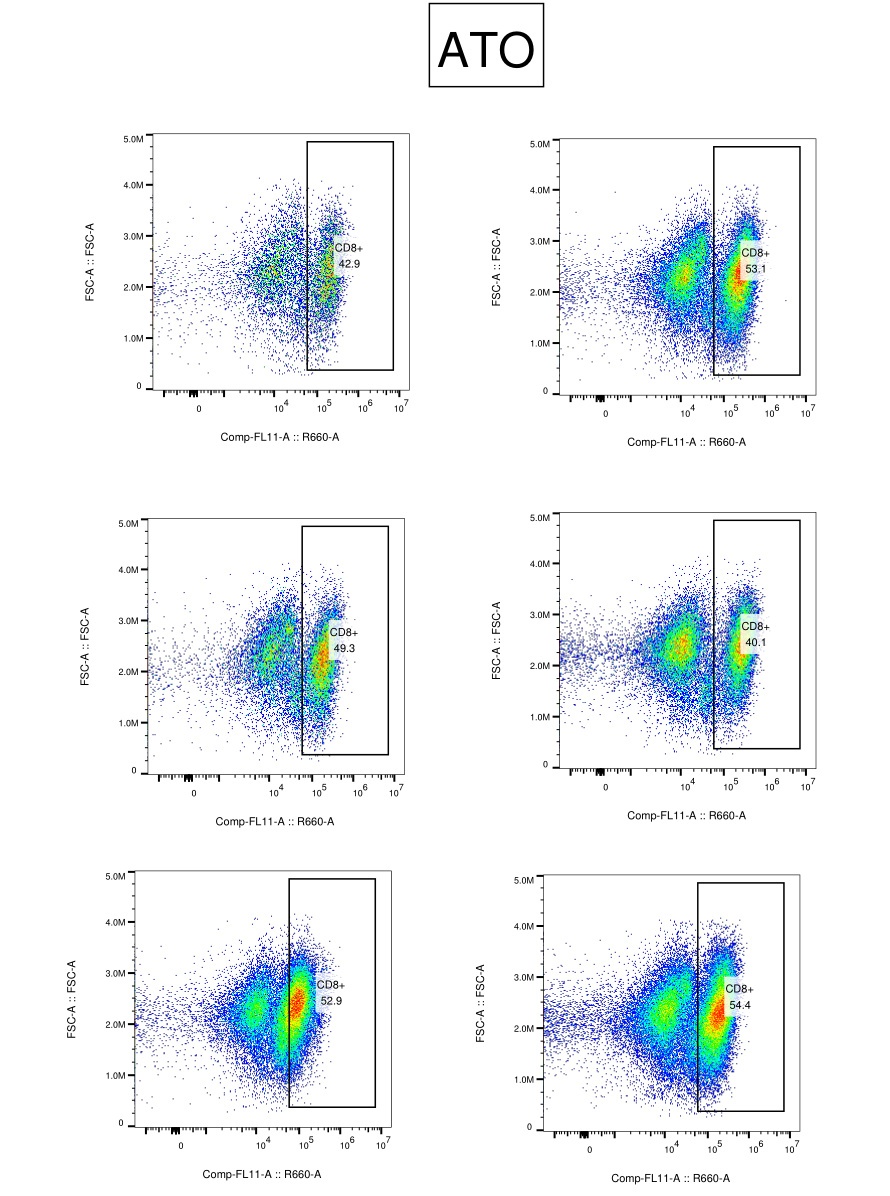


Experimental data of CD8^+^ T cells-ATO group


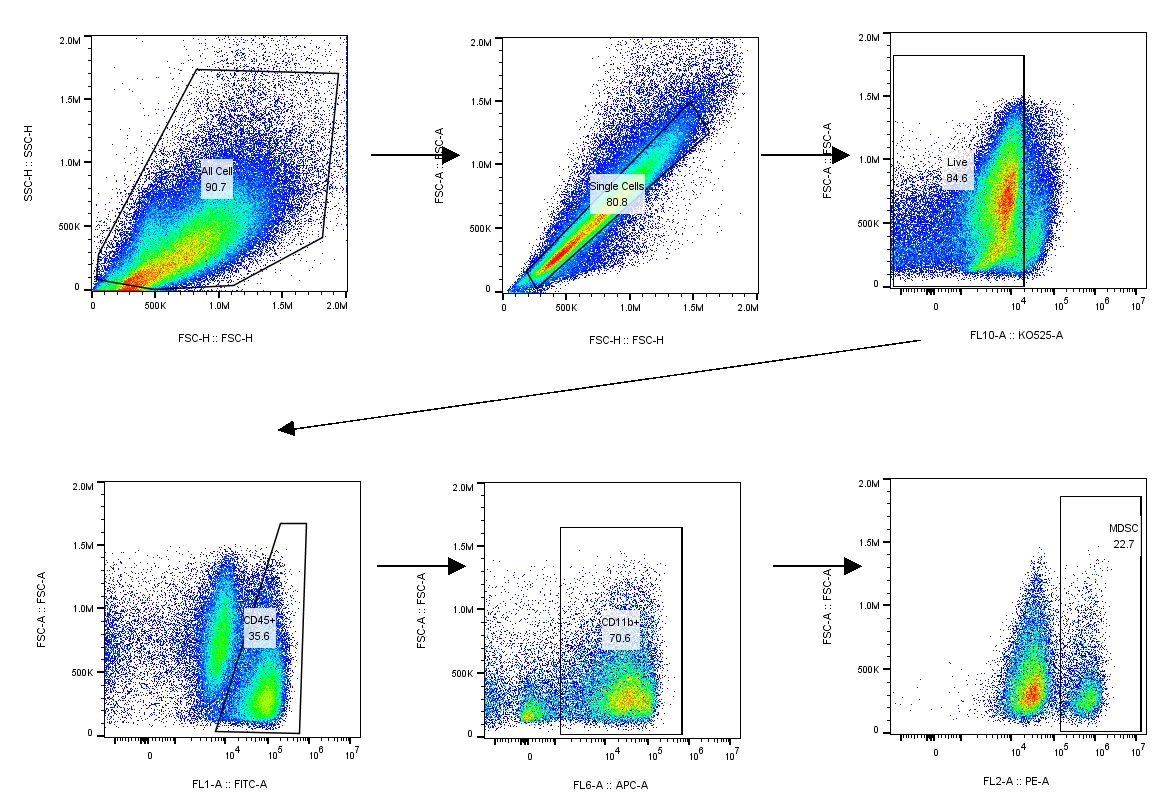


Gating strategy of MDSC


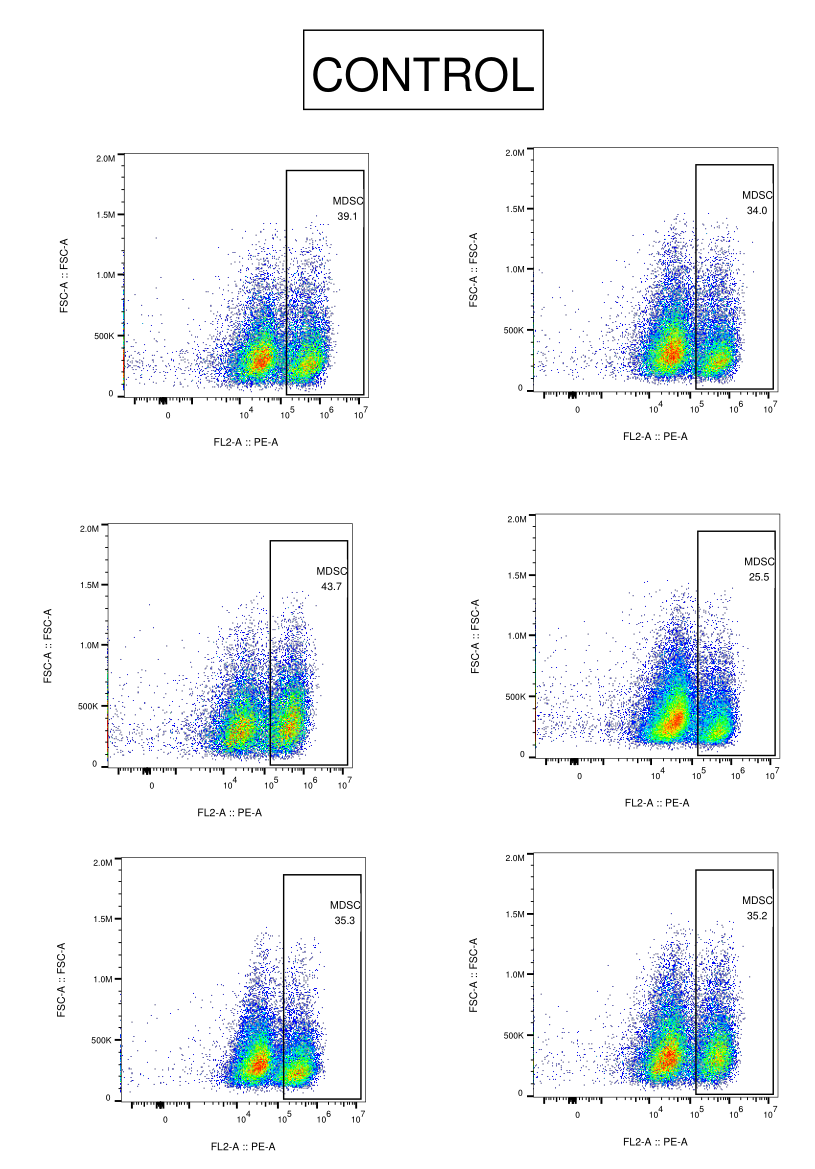


Experimental data of MDSC-CONTROL


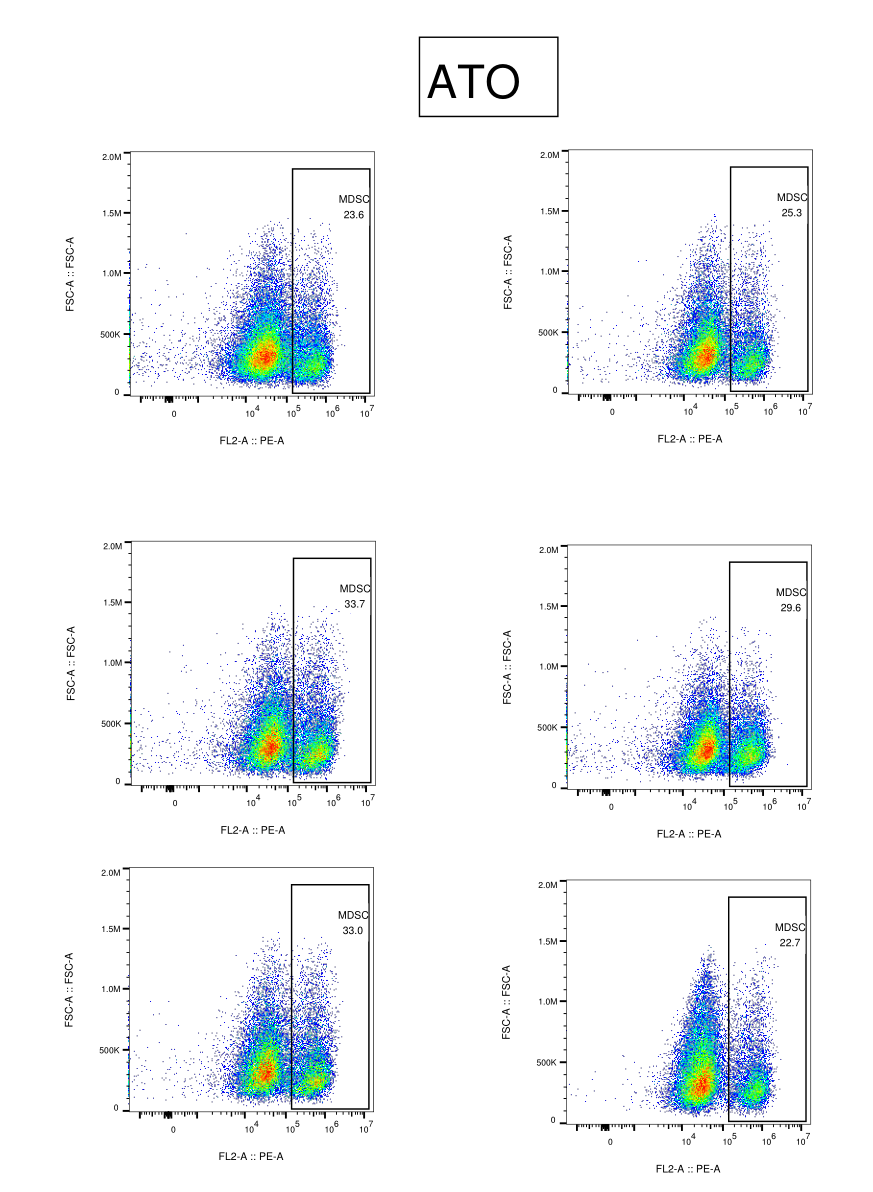


Experimental data of MDSC-ATO
